# Establishment of an Immunological Method for Detection of Bluetongue Virus by Fluorescence-Linked Immunosorbent Assay

**DOI:** 10.1128/spectrum.01429-22

**Published:** 2022-09-26

**Authors:** Jiajia Yin, Aiping Wang, Jingming Zhou, Yumei Chen, Chao Liang, Xifang Zhu, Ying Zhang, Yankai Liu, Rui Jia, Gaiping Zhang

**Affiliations:** a School of Life Sciences, Zhengzhou University, Zhengzhou, People’s Republic of China; Barnard College, Columbia University

**Keywords:** bluetongue virus, quantum dots, fluorescence-linked immunosorbent assay

## Abstract

Bluetongue (BT) is a severe noncontagious infectious disease that occurs in sheep and wild ruminants but occasionally also in cattle and camels. The worldwide BT pandemic has had a significant impact on global livestock production. Rapid detection helps prevent outbreaks of bluetongue disease. Fluorescence-linked immunosorbent assay (FLISA) labeled with quantum dots (QDs) is typically used for detection due to its high sensitivity. There has been no reported detection of BT virus (BTV) using QD-based fluorescence immunoassays. In this study, monoclonal antibodies (MAbs) against BT were prepared by immunizing BALB/c mice with recombinant VP7 protein. Two MAbs with high sensitivity and specificity were selected as the detection antibody (2F11) and capture antibody (11B7). Then, the detection antibody was coupled with QDs to prepare QD-MAb fluorescence probes. Fluorescence-linked immunosorbent assay is highly specific, detecting only VP7 protein/BTV, and did not show any nonspecific reactions with other reoviruses. The detection limit of VP7 protein was 3.91 ng/mL using fluorescence-linked immunosorbent assay, with a coefficient of variation (CV) of less than 15%. The establishment of rapid, sensitive direct FLISA has potential for bluetongue virus detection and control of BT vaccine quality.

**IMPORTANCE** Bluetongue virus causes the severe infectious disease BT. BTV has many serotypes, and there is no cross-protection among different serotypes. BT is listed as a notifiable animal infectious disease by the World Organisation for Animal Health (OIE) and occurs throughout the world, causing significant economic losses. The establishment of a fast and effective detection method is the key to controlling and preventing this disease. Current methods for detecting BTV mainly include reverse transcription-PCR (RT-PCR), enzyme-linked immunosorbent assays (ELISA), and immunochromatographic strips that are based on antigen-antibody recognition. Immunoassays are most commonly used because of their low cost, high specificity, and fast analysis, making them particularly useful for routine monitoring. These conventional detection strategies for BTV have some drawbacks. Recently, FLISA has been drawing attention due to its sensitivity, which is higher than traditional immunoassays. Fluorescence-linked immunosorbent assays (FLISA) using fluorescent materials as labels overcome ELISA’s disadvantage of being time-consuming.

## INTRODUCTION

A severe noncontagious infectious disease, bluetongue (BT) is caused by the bluetongue virus (BTV), which commonly affects sheep and wild ruminants and occasionally also cattle and camels (1). Symptoms of the infection include fever, cyanotic tongue, ulcers in the mouth and stomach, and even death for the animals (2). Bluetongue disease was named after it was confirmed by the scientist A. Theiler in 1905. BT is listed as a notifiable animal infectious disease by the World Organisation for Animal Health (OIE) and occurs around the world, causing significant economic losses (3, 4).

Until now, 28 serotypes (BTV1 to BTV28) of BTV were recognized with no cross-protection between any two serotypes (5). It is imperative to establish a rapid detection method to provide fast, safe, and effective technical means for epidemiological investigation and surveillance in China. Currently, the commonly used laboratory diagnostic methods for BTV mainly include molecular biology and serological detection. The molecular biological detection methods comprise reverse transcription-PCR (RT-PCR) and fluorescent quantitative PCR (6). Both have good accuracy and sensitivity but require expensive instruments to operate. The conventional serological methods for BTV detection include indirect enzyme-linked immunosorbent assay (ELISA), competitive ELISA, indirect immunofluorescence assay (IFA), and virus neutralization assay. At present, competitive ELISA is more commonly used; it has strong specificity and sensitivity but is time-consuming (7).

Rapid antigen detection (RAD) tests play a key role in reducing the spread of disease and could be a potential tool in early BTV diagnosis (8). Currently, nanomaterials are widely used in the rapid detection of human and animal diseases. For example, quantum dots (QDs) combined with CdSe/ZnS, which shows beneficial characteristics such as strong fluorescence intensity, good photochemical stability, multiple long-term excitations, and resistance to quenching, has been successfully used in qualitative and quantitative analysis of biological samples (9–11). Therefore, this study was designed to prepare direct fluorescence immunoassays for the screening of bluetongue disease.

Bluetongue virus is a double-stranded RNA virus made up of 10 linear gene segments, which encode 7 structural proteins (VP1 to VP7) and 4 nonstructural proteins (NS1 to NS4) (7, 12). The VP7 proteins of all serotypes of BTV carry conserved lysine residues, indicating that the structural protein VP7 is conserved in different serotypes of BTV. VP7 contains BTV group-specific antigenic determinants, which can stimulate the body to produce a strong group-specific immune response. The humoral immune response induced by VP7 is stronger than the cell-mediated immune response, so VP7 is widely used in BTV serological diagnosis (13).

As part of this study, VP7 protein was used as an immunogen in order to obtain monoclonal antibodies (MAbs) against BTV. A fluorescence-linked immunosorbent assay (FLISA) for BTV was then developed using two VP7-specific MAbs, which provides new technical means for the rapid screening and control of bluetongue disease.

## RESULTS

### Production and purification of BTV1 recombinant VP7.

**FIG 1:**
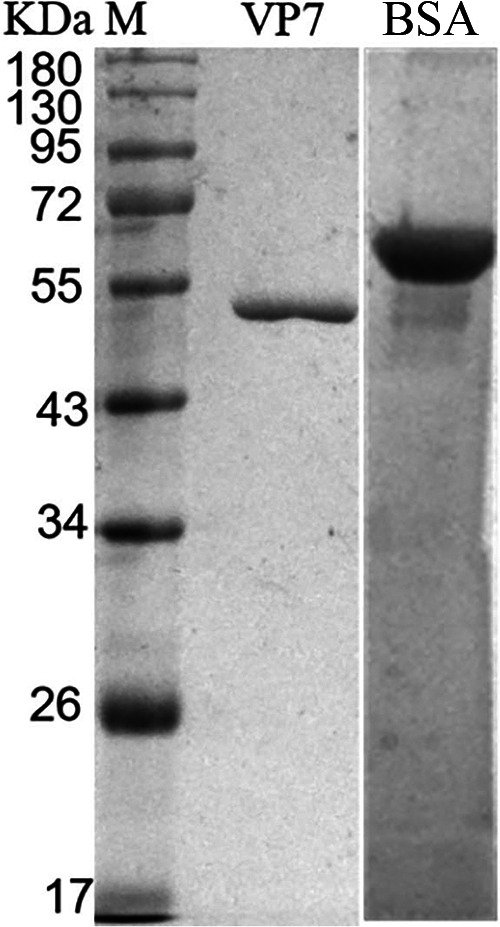
SDS-PAGE of VP7 protein and BSA. Lanes: M, protein molecular weight marker; VP7, VP7 protein; BSA, bovine serum albumin.

Recombinant VP7 was produced using the BL21(DE3) prokaryotic cell expression system. SDS-PAGE analysis indicated that the protein was eluted in phosphate buffer (PB) containing 100 mM imidazole, and the purity was >90%, paving the way for it to be immunized in the next step. The concentration of VP7 protein was measured using NanoDrop 2000 protein measurements and by SDS-PAGE compared with quantitative bovine serum albumin (BSA) (1 μg/μL); the protein concentration of VP7 was 0.1 mg/mL (Fig. 1).

### Preparation of monoclonal antibodies.

**FIG 2:**
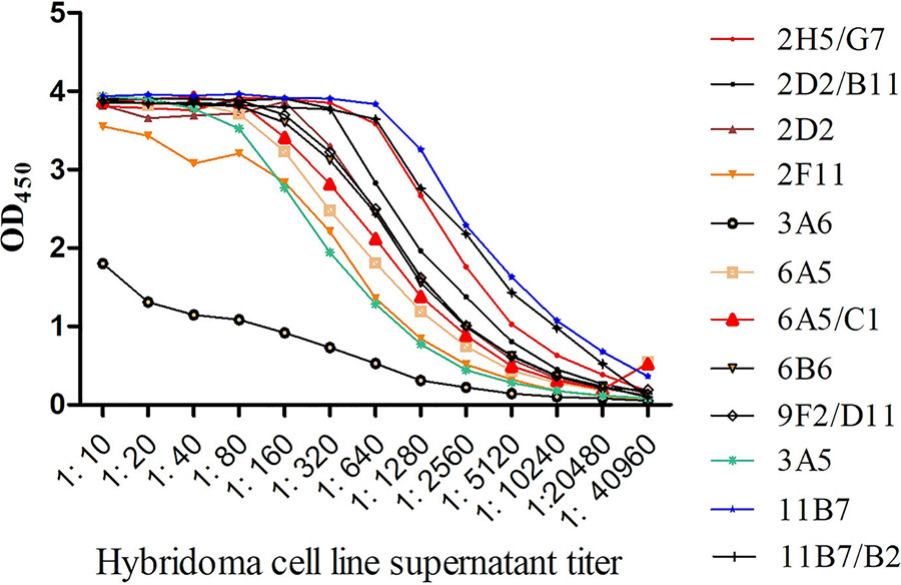
Titers of supernatant of 12 hybridoma cell lines. OD_450_, optical density at 450 nm.

A serum sample was collected from the tails of mice before the cells were fused in order to determine the antibody titer. ELISA analysis revealed a titer of up to 1:102,400 in the mice serum. The spleen cells of the mice with the highest titer fused with Sp2/0 myeloma cells in this study. Twelve MAbs against VP7 were generated in this study, and ELISA titers of the supernatants of 12 hybridoma cell lines ranged from 1:2,560 to 1:40,960 (Fig. 2).

### Characterization of the supernatants of the hybridoma cell lines.

**FIG 3:**
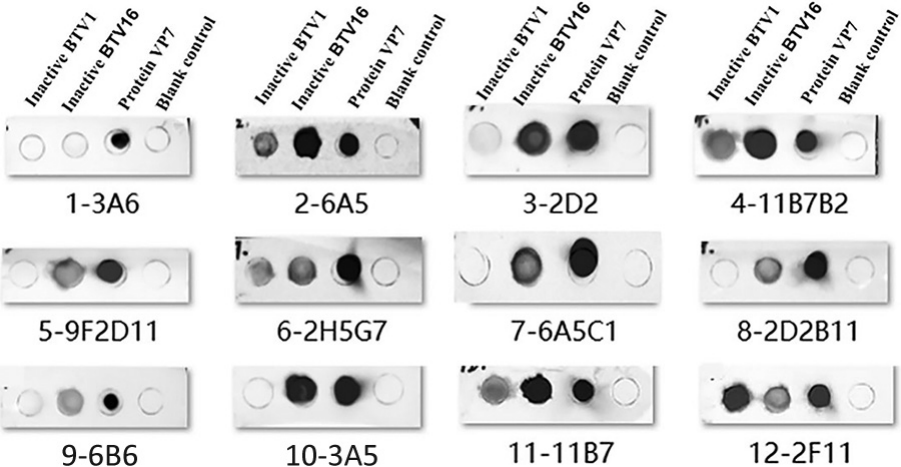
Results of dot-ELISA analysis of the reactivity of hybridoma cell line supernatants with BTV.

The reactivity of the hybridoma cell line supernatants with BTV was assessed using dot-ELISA. Nitrocellulose (NC) filter membranes were coated with inactivated BTV1, inactivated BTV16, and recombinant VP7 protein. As the primary and secondary antibodies, respectively, supernatant and 5,000-fold diluted horseradish peroxidase (HRP)-conjugated goat anti-mouse immunoglobulin (IgG) antibody were used. The dot-ELISA results indicated that the hybridoma cell line supernatants specifically reacted with inactivated BTV1, inactivated BTV16, and recombinant protein VP7, except for the supernatants of 1-3A6 and the control group, which had no response (Fig. 3).

### Purification of monoclonal antibodies.

**FIG 4:**
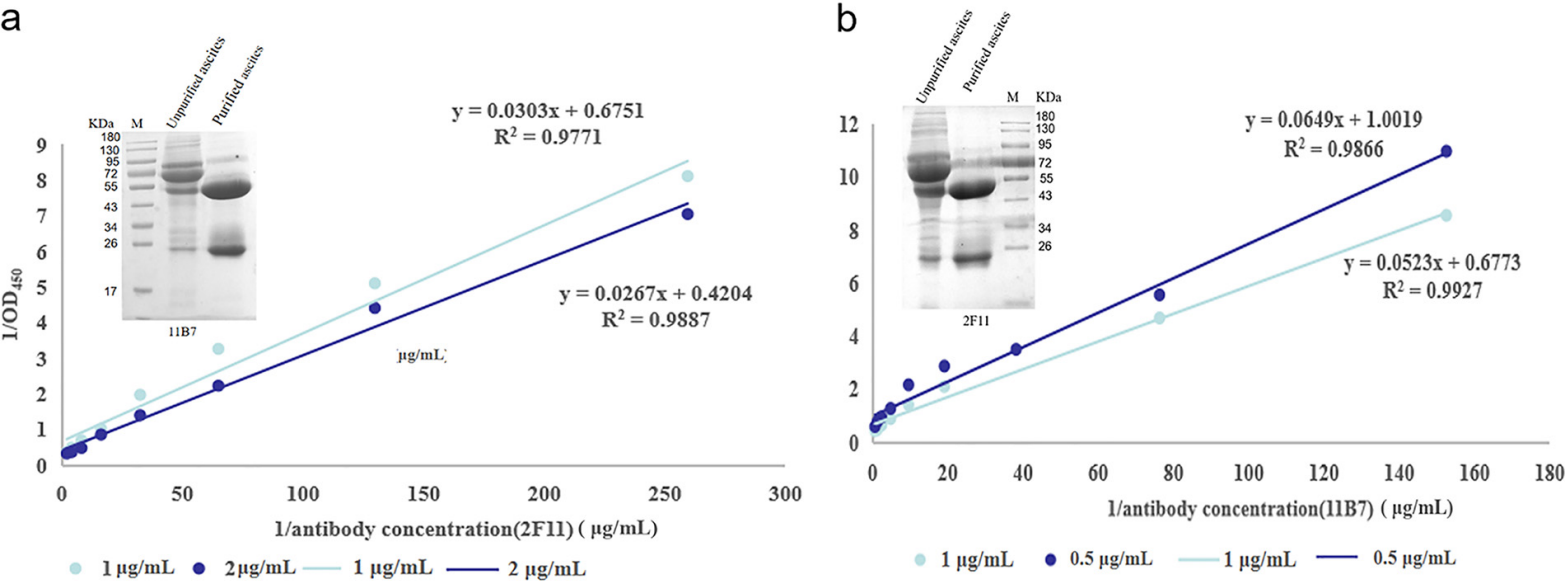
Purification and identification of monoclonal antibodies of 11B7 (a) and 2F11 (b).

Two hybridoma cell lines that reacted well with BTV were selected for purification. Thereafter, the ascites liquid was collected and purified by octanoic acid and ammonium sulfate precipitation. The purification results are shown in Fig. 4. The affinity of the two monoclonal antibodies was 4.32 × 10^7^ (2F11) and 2.05 × 10^8^ (11B7), respectively, so 2F11-MAb was used as the capture antibody.

**FIG 5:**
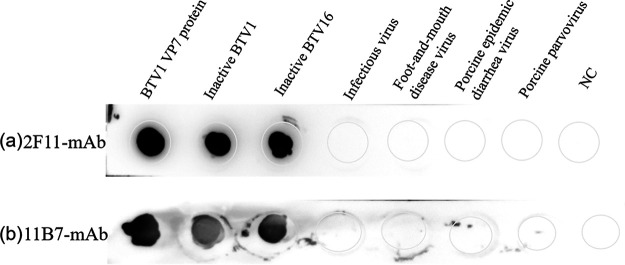
Specificity evaluation of 2F11-MAb (a) and 11B7-MAb (b) with (left to right) the BTV1 VP7 protein produced in this study; inactive BTV1; inactive BTV16; infectious bronchitis virus; foot-and-mouth disease virus; porcine epidemic diarrhea virus; porcine parvovirus; negative control (NC).

**(i) Specificity of MAbs.** To identify the specificity of MAbs, the VP7 proteins of BTV, inactivated BTV1/16, infectious bronchitis virus (IBV), foot-and-mouth disease virus (FMDV), porcine parvovirus, and porcine epidemic diarrhea virus were simultaneously detected using dot-ELISA. The results showed that the MAbs specifically reacted to inactivated BTV and recombinant VP7 protein, whereas they had no response to the other diseases (Fig. 5).

**FIG 6:**
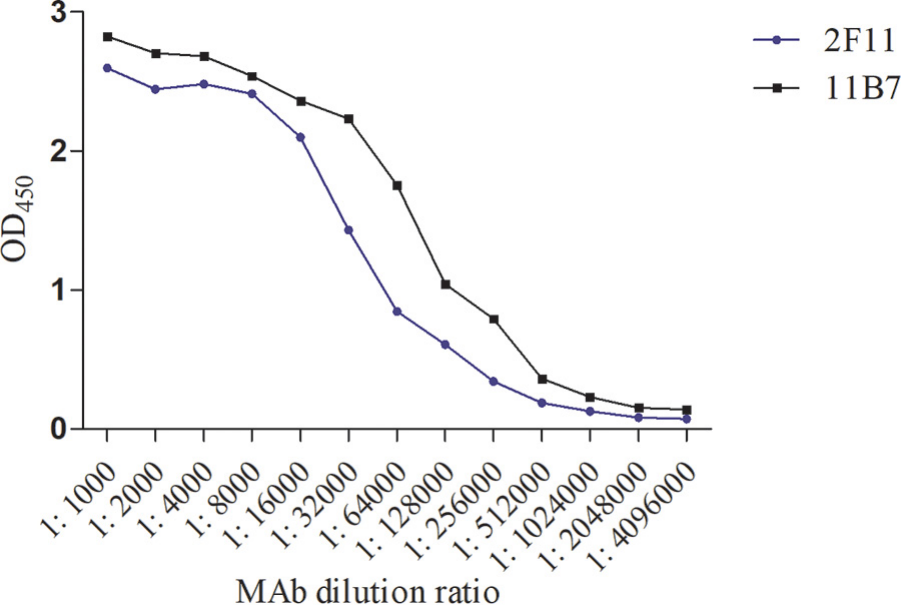
Titer assay of monoclonal antibodies.

**(ii) Titers of MAbs.** Indirect ELISA was used to determine the MAb sensitivity by using recombinant VP7 protein as the immobilized antigen. At a wavelength of 490 nm, the results of indirect ELISA were determined on a microplate reader (BioTek Instruments, Inc., Winooski, VT, USA). The sensitivities of the monoclonal antibodies 11B7 and 2F11 were 1:2,048,000 and 1:1,024,000, respectively (Fig. 6).

### Identification of the fluorescent probes.

**FIG 7:**
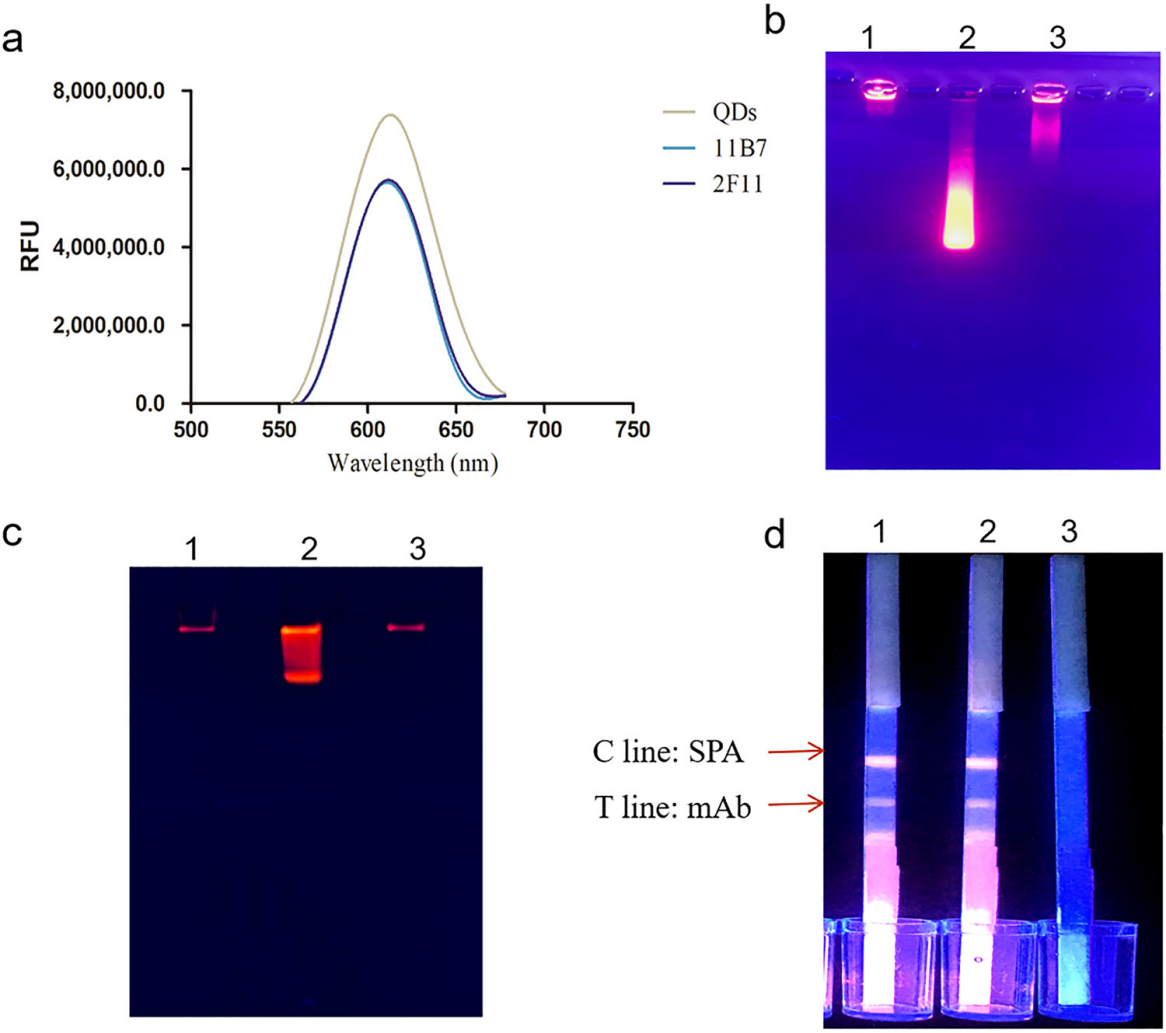
Characterization of the QDs and conjugated MAb-QDs. (a) Fluorescence emission spectrum of orange-red QDs and anti-VP7-MAb-QDs (excitation wavelength, 450 nm; emission peak wavelength, 610 nm). (b) Agarose gel electrophoresis and (c) SDS-PAGE. Lanes: 1, 2F11-MAb-QD; 2, orange-red QDs; 3, 11B7-MAb-QD. (d) Biological activity. Lanes: 1, 2F11-MAb-QDs; 2, 11B7-MAb-QDs; 3, orange-red QDs. RFU, relative fluorescence units.

The carboxyl groups on the surface of water-soluble QDs can be activated by carbodiimide hydrochloride (EDC) and react with the amino groups of VP7 MAbs to form QD-MAb conjugates. As described by Yao et al., fluorescence measurements, agarose gel electrophoresis, and SDS-PAGE were used to characterize the immunofluorescence probes. Fluorescence spectra of the MAb-QDs and orange-red QDs are shown in Fig. 7a. The fluorescence emission peaks of the MAb-QDs did not shift significantly compared with those of the free QDs, which is consistent with previous research (14). Orange-red QDs and anti-VP7-MAb-QDs emitted at 610 nm, but the MAb-QDs produced fluorescence weaker than that produced by the free QDs. A possible explanation would be that immobilized antibodies influence fluorescence detection or quench fluorescence during coupling, indicating that the MAbs have been successfully connected.

Due to the natural characteristics of the specific binding between antibodies and antigens, immunochromatography is a simple and visual method for detecting conjugates and biological activities. Briefly, based on the immunochromatographic assay (ICA) principle, probe solution was forced by capillary action along the NC membrane upward from the suction pad past the MAb-QD probe, the corresponding antigen (MAb), and the staphylococcal protein A (SPA), control (C), and test (T) lines. As shown in Fig. 7d, the MAb-QDs 11B7 and 2F11 emitted bright fluorescent bands on the C and T lines when irradiated with a handheld UV lamp, whereas the control showed no fluorescence, illustrating that the MAb-QDs retained biological activity.

### Specificity evaluation of FLISA.

**FIG 8:**
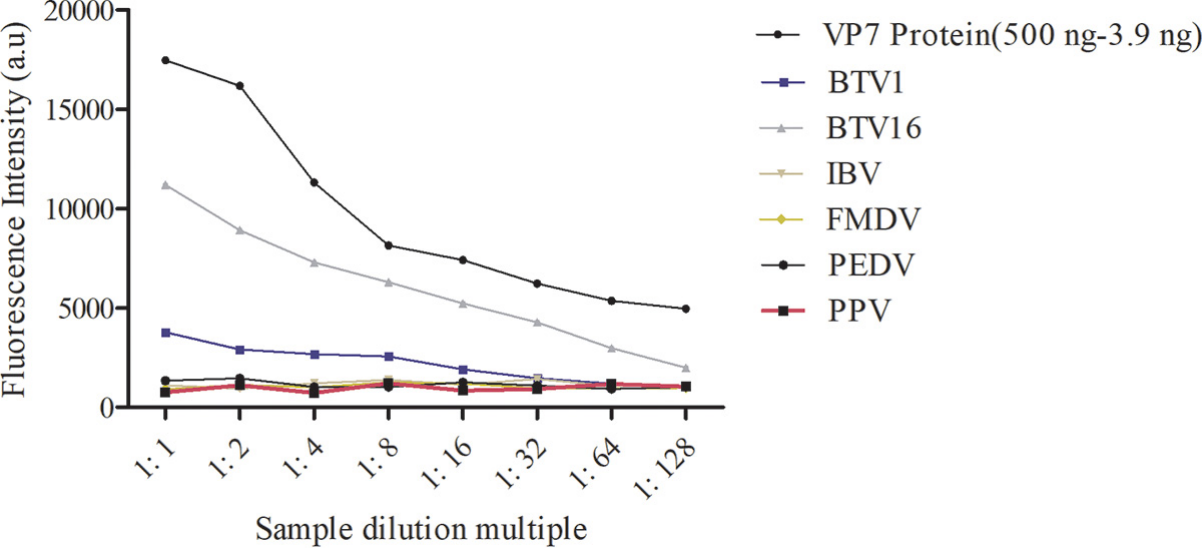
FLISA specificity evaluation. a.u, arbitrary units.

The double-antibody sandwich mode fluorescence immunosorbent assay was established using the VP7-specific monoclonal antibodies 2F11 and 11B7 as conjugated and captured antibodies, respectively. The fluorescence intensity results showed that only the VP7 protein produced in this study and inactivated BTV had obvious fluorescence values, whereas infectious bronchitis virus (IBV), foot-and-mouth disease virus (FMDV), and other viruses did not show any specific reactions (Fig. 8). This indicates that FLISA had high specificity for the detection of BTV.

### Sensitivity evaluation.

**FIG 9:**
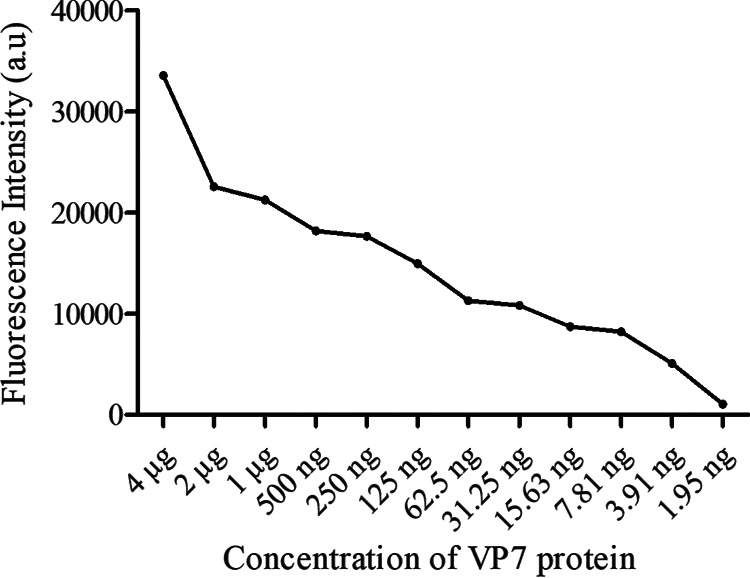
FLISA sensitivity evaluation.

To test the sensitivity of the FLISA assay, serial dilutions of VP7 protein were used, ranging from 4,000 to 1.95 ng/mL. The fluorescence intensity decreased as the protein concentration was reduced, and the detection limit of VP7 was 3.91 ng/mL (Fig. 9).

### Repeatability evaluation.

**FIG 10:**
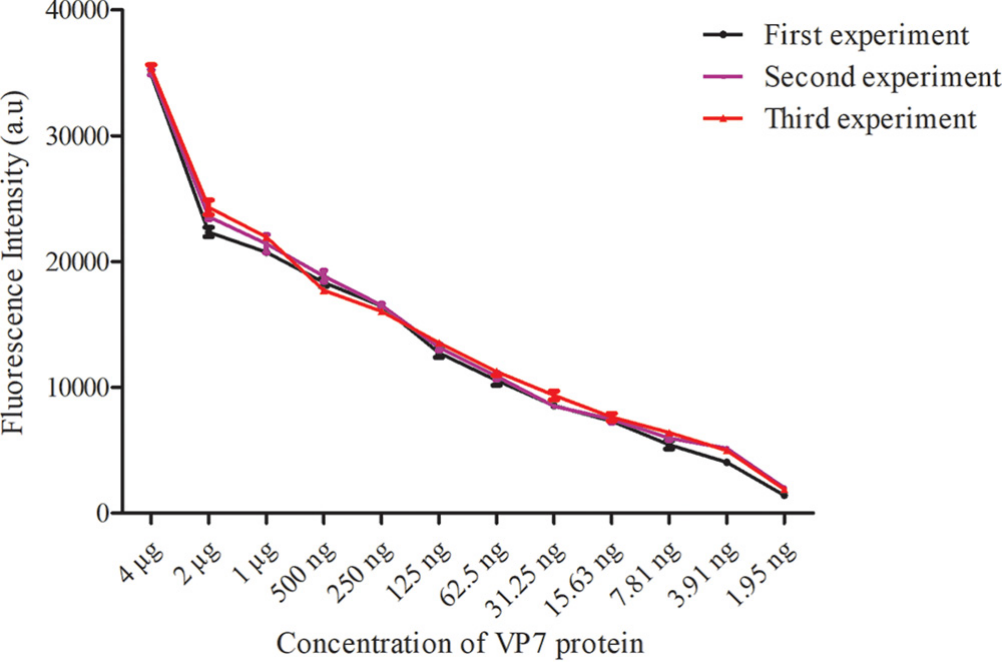
FLISA repeatability analysis.

Using serially diluted VP7 protein as the sample for FLISA evaluation, three independent experiments were conducted by different operators at different times. The highest CV values of the three experiments were 10.41%, 6.97%, and 9.20%, lower than the average CV (15%), which indicates that the reproducibility of this experiment is good (Fig. 10).

## DISCUSSION

BT is an infectious disease caused by the bluetongue virus, which has numerous serotypes with no cross protection. The OIE lists BT as a notifiable animal infectious disease that causes significant economic losses throughout the world (3, 15). The establishment of a fast and effective detection method is the key to controlling and preventing this disease. The current methods for detecting BTV mainly include RT-PCR (16), ELISA (17–19), and immunochromatographic strips that are based on antigen-antibody recognition (20). Immunoassays are most commonly used because of their low cost, high specificity, and fast analysis, making them particularly useful for routine monitoring (21). These conventional detection strategies for BTV have some drawbacks; for example, RT-PCR requires large-scale experiments and is costly, ELISA is time-consuming, and the colloidal gold strip flux is not high. Recently, fluorescence-linked immunosorbent assay (FLISA) has been drawing attention because it has higher sensitivity than traditional immunoassays. FLISA using fluorescence materials as labels overcomes ELISA’s disadvantage of being time-consuming (22).

BTV VP7 resides on the outer surface of the core particles and contains BTV group-specific epitopes, which can trigger a strong antibody response and are often used to detect viral infections (23). It has been reported that serological detection of BTV is mainly based on VP7 antibody detection (24, 25). BTV antigen detection mainly employs the antibody of VP7-based sandwich ELISA (26, 27). Prokaryotic expression has the advantages of simplicity, ease of purification, and mass production as a mature expression system (28). In recent years, QDs have become an ideal label for fluorescence immunoassays due to their higher photochemical stability than traditional organic fluorophores (29).

In this study, a total of 12 anti-BTV1 VP7 monoclonal antibodies were prepared using BTV1 VP7 protein expressed in the prokaryotic expression system. Two MAbs, 2F11 and 11B7, were selected as conjugation and capture antibodies for FLISA using dot-ELISA. In addition to basic immunology studies, the remaining 10 MAbs are also useful for pathogen detection, antigen purification, and disease diagnosis and prevention. An immunological assay’s performance is heavily influenced by the biological characteristics of monoclonal antibodies. The specificity and sensitivity of the two monoclonal antibodies used for detection were identified. They displayed specific reactivity with BTV-related antigen and with titers of up to 1:2,048,000, indicating high specificity and sensitivity of the two MAbs. The quantum dot was used to establish a fluorescence immunoassay for BTV and was able to specifically distinguish VP7 and inactivated BT viruses. The detection of VP7 antigen was as low as 3.91 ng/mL, with good repeatability. However, due to the limitation of the sample, only the existing inactivated virus was tested in this study, and the feasibility of the FLISA method was primarily verified.

In this study, for the first time, QDs were used in a fluorescence immunoassay for BTV detection. Compared with the traditional immunoassay, FLISA offers a novel method for BTV detection. As a diagnostic tool, it may not have the same sensitivity as molecular detection, such as RT-PCR. However, FLISA was able to detect luminescence immediately after removing free binding molecules, and no secondary antibody or chromogenic substrate was required, so the incubation step was omitted and the analysis time was shortened, making FLISA economical for routine detection. Moreover, the sensitivity of FLISA can be further improved by modifying the materials and optimizing the conditions.

This study provides a new tool for fast and accurate BTV detection that can be further applied to BTV surveillance activities.

## MATERIALS AND METHODS

### Materials and apparatus.

Inactivated viruses 1 and 16 were kindly provided by the Yunnan Academy of Animal Husbandry and Veterinary Sciences. Goat anti-mouse IgG HRP conjugate was obtained from Beijing Solarbio Science & Technology Co., Ltd. (Beijing, China). ZnCdSe/ZnS (core/shell) carboxylated orange-red QDs (emission wavelength [λem], 610 nm; particle size, 24 nm) were purchased from JiaYuan Quantum Dots Co., Ltd. (Wuhan, China). Freund’s complete and incomplete adjuvants, hydroxysuccinimide (NHS), 3,3,5,5-tetramethylbenzidine (TMB), carbodiimide hydrochloride (EDC), polyethylene glycol (PEG) 1500, fetal bovine serum (FBS), and 1640 medium were obtained from Sigma-Aldrich Chemical Co. (St. Louis, MO, USA). Staphylococcal protein-A (SPA) was purchased from Aladdin (Shanghai, China). The rest of the chemicals and organic solvents were analytical grade or higher.

Opaque black plates were obtained from CoStar, Inc. (Cambridge, MA, USA). The gel imaging system (Gel Doc TM XR+) and electrophoresis instrument (MiniPROTEAN Tetra System) were both manufactured by Bio-Rad (CA, USA). SDS-PAGE electrophoresis and NanoDrop spectrophotometry were performed using a gel imaging system purchased from Bio-Rad (CA, USA). SpectraMaxi3 was purchased from Migu Molecular Instruments Co. Ltd. (Shanghai, China).

### Expression and purification of recombinant VP7 protein.

A pair of BTV1 VP7 primers (synthesized by Sangon Biotech, China) were designed according to the complete gene sequence of VP7 (GenBank accession number KF664129.1) and used to amplify the VP7 gene. The amplified VP7 gene was cloned into the pET-32a vector to construct the recombinant plasmid vector pET-32a-VP7. Then, pET-32a-VP7 was transformed into Escherichia coli BL21(DE3) competent cell lines to induce the expression of recombinant VP7 protein. The recombinant VP7 protein was purified using Ni affinity chromatography (GE Healthcare, USA) in a buffer (20 mM PB, 150 mM NaCl, pH 7.0) according to the manufacturer’s instructions. The recombinant VP7 protein was analyzed using SDS-PAGE, and the concentration of VP7 protein was measured.

### Generation of monoclonal antibodies.

Briefly, 6-week-old female BALB/c mice (*n* = 2) were immunized with the VP7 protein produced in this study at a dose of 20 μg for each mouse. For the first vaccination, Freund’s complete adjuvant and recombinant VP7 protein were used, while the second and third immunizations were administered with Freund’s incomplete adjuvant. Vaccinations were administered every 3 weeks, and blood samples were collected 14 days after the third immunization. The antiserum titer was determined by ELISA. The mouse with the highest titer was selected for superimmunization, followed by cell fusion. The mouse spleen was aseptically ground, then fused with Sp2/0 myeloma cells using PEG 1500 at a ratio of 8:1. ELISA was used to screen the hybridoma cells, and then limiting dilution was used to obtain a monoclonal culture that produced antibodies stably. Two positive hybridomas, 11B7 and 2F11, were each injected into the abdomen of one of two BALB/c mice by intraperitoneal injection to produce ascitic fluid. The fluid was collected and purified by octanoic acid and ammonium sulfate precipitation. Finally, the specificity and titer of the obtained MAbs were evaluated.

### Production and analysis of QD-MAb conjugates.

Following Liu et al. (30), immune fluorescent probes were synthesized based on the EDC method, with slight modifications. Briefly, 1.25 μL (8 μM) of QD610 and 3.38 μL of EDC (1 mg/mL dissolved in a boric acid solution at pH 8.0) were stirred for 30 min at 25°C. Subsequently, 23.58 μL MAb (11B7) was added to the above solution and incubated at 25°C for 3 h. Then, 10% BSA was added to block the unbound site with oscillation for 30 min. Finally, the solution was centrifuged at 11,833 × *g* for 3 min to remove agglomeration. Conjugates were transferred to microcentrifuge tubes to be stored away from light. The method for preparation of the QD-MAb (2F11) probes was similar to the above method. The conjugates were assessed using nondenatured polyacrylamide gel electrophoresis (native PAGE) and nondenatured agarose gel electrophoresis-immunochromatographic analysis.

### FLISA procedure.

We depict the process of using QDs in FLISA. To perform the experiment, 50 μL MAb (11B7) (2 μg/mL) was diluted in sodium carbonate-sodium bicarbonate solution (CBS; pH 9.6) and added to a 96-well opaque black microtiter plate, incubated at 37°C for 2 h or overnight at 4°C. The microplate was then lightly washed with PBST (1× phosphate buffered saline containing 0.05% Tween 20). In order to block nonspecific and unreacted sites, 200 μL of pig serum (5%, wt/vol) were added to each microplate well for 1 h at 37°C. The microporous plate was washed with PBST 3 times.

Immediately after adding the sample, the QD-MAb mixture at the optimal concentration (1:100) was added to the wells, and the plate was incubated for 1 h at 37°C. Following incubation, the microplate was rinsed five times. A SpectraMaxi3 microplate reader was used to measure the fluorescence intensity at 610 nm and the excitation wavelength of 450 nm.

**(i) Specificity evaluation.** MAb (11B7) (2 μg/mL) was coated onto each well of an opaque black microtiter plate. Subsequently, BTV1 VP7 protein, inactivated BTV1, inactivated BTV16, infectious bronchitis virus (IBV), foot-and-mouth disease virus (FMDV), porcine parvovirus (PPV), and porcine epidemic diarrhea virus (PEDV) were each added to separate microtiter wells. QD-MAb diluted in PBS at the optimal dilution (1:100) was then added and incubated at 37°C for 1 h. The fluorescence intensity was measured using a SpectraMaxi3 microplate reader.

**(ii) Sensitivity evaluation.** To determine the sensitivity of FLISA, the BTV1 VP7 protein (0.1 mg/mL) in this study was 2-fold diluted with 0.01 M PBS in a gradient from 4,000 ng/mL to 1.95 ng/mL. An aliquot (50 μL) of each sample was added to the 96-well opaque black microtiter plate and incubated with QD-MAb (11B7) at the optimal dilution (1:100) for 1 h at room temperature. The fluorescence intensity was measured using a SpectraMaxi3 microplate reader and compared with that of the control group.

**(iii) Repeatability evaluation.** We evaluated the repeatability of FLISA using continuous dilution of BTV1 VP7 protein. On different days, three experiments were conducted, and each sample in each experiment was repeated three times. The repeatability was analyzed by measuring the coefficient of variation (CV).

### Ethics approval.

Experimental research protocols for the production of monoclonal antibodies in mice were conducted according to animal care guidelines issued by the Chinese Council on Animal Care. The research protocol was approved by the Joint Unit of Zhengzhou University and the Animal Ethics Review Committee of the Key Laboratory of Animal Immunology, Henan Academy of Agricultural Sciences (LLSC410220029), in accordance with its policies.
